# Envisioning patient safety in Telehealth: a research perspective

**DOI:** 10.1007/s12553-014-0078-7

**Published:** 2014-04-10

**Authors:** José Luis Monteagudo, Carlos H. Salvador, Luis Kun

**Affiliations:** 1 Telemedicine and e-Health Unit, Carlos III Institute of Health, Madrid, Spain; 2 William Perry Center for Hemispheric Defense Studies of the National Defense University, Washington, DC USA; 3 Unidad de Investigación en Telemedicina y e-Salud, Instituto de Salud Carlos III, Pabellón 14, Avda. Monforte de Lemos, 5, 28029 Madrid, Spain

**Keywords:** Healthcare, Telehealth, Patient safety, Complex socio-technical systems

## Abstract

This article explores the need for research into patient safety in large-scale Telehealth systems faced with the perspective of its development extended to healthcare systems. Telehealth systems give rise to significant advantages in improving the quality of healthcare services as well as bringing about the possibility of new types of risk. A theoretical framework is proposed for patient safety for its approach as an emerging property in complex socio-technical systems (CSTS) and their modelling in layers. As regards this framework, the differential characteristic Telehealth elements of the system have been identified, with a greater emphasis on the level of Telehealth system and its typical subsystems. The bases of the analysis are based on references in the literature and the experience accumulated by the researchers in the area. In particular, a case describing an example of Telehealth to control patients undergoing treatment with oral anticoagulants is used. As a result, a series of areas of research into and topics regarding Telehealth patient safety are proposed to cover the detectable gaps. Both the theoretical and practical implications of the study are discussed and future perspectives are reflected on.

## Introduction

### Telehealth and the transformation of healthcare systems

Telehealth is currently one of the fields of greatest growth associated with the implementation of new models for attention to chronic patients in order to improve the quality of the services whilst favoring the sustainability of the social and healthcare systems. The name Telehealth encapsulates a wide range of distance healthcare services and applications using Information and Communications Technologies (ICT) for home care, long-term care, prevention, health promotion, self-care and support for the integration of social and healthcare services [1–4]. Telehealth is also useful for in-time early detection and rapid intervention by the health authorities using these new tools for the monitoring the health of the population [5, 6]. With the help of personal biomedical devices and wearable sensors, Telehealth applications may be used for tele-monitoring the state of the patents whilst, at the same time, increasing their adherence to their treatment [7–11]. In this way the patients are able to transmit information on their functional state, quality of life and vital signs to the healthcare provider so that they can analyze the data and work on them as a consequence. Furthermore, Telehealth systems normally offer functionalities to help the patients follow and understand this information so that they can be better informed as to the state and evolution of their condition. Furthermore, Telehealth tools open up new channels of communications between patients, providers, family, friends, and community organizations in order to improve health, and share information and vital experiences. On the other hand, Telehealth systems are a highly beneficial resource in emergency situations. Simply consider the potential of getting round the problems detected in the Fukushima tragedy when the clinical records and the medication treatments of the patients were lost [12].

Web technologies have been used to implement patient portals that offer the patients online access to their personal electronic health records (PHRs) and tools to help with the management of their chronic conditions for a long time. These portals also facilitate communication between patients and healthcare professionals, as well as access to educational materials to facilitate care and the promotion of health.

The patient portals have become popular in recent years since being implemented by a large number of healthcare organizations for their community of users. Many people use social networks to obtain information on their state of health and communicate it effectively with their healthcare providers as well as identifying other people with similar chronic conditions with whom they can share information and get support as well as being able to identify healthcare educational resources [13, 14]. On the other hand, a market oriented at consumers has recently emerged based on smart phones and tablets using email, SMS messaging and mobile Internet. There are a growing number of Apps that can be directly downloaded for their use by the patients and carers in general [15].

Telehealth is included within the general domain of eHealth and is related to other terms such as personal health, mHealth, ubiquitous health, health 2.0 and connected health, which are frequently overlapped and exchanged in the literature. Since the last decade of the 20th century, a large number of Telehealth pilot projects and demonstrators have come about. There is a growing activity currently in the deployment of regional and national networks, especially directed to the attention to chronic patients, fragile elderly people and dependents including support for self-care and for the prevention and promotion of health. Notable examples include the case of the Telehealth network for the Veterans Administration in the USA [16]; the 3 Million Lives project in the UK [17] and the EIP-AHA (European Innovation Partnership on Active and Healthy Ageing) initiative of the European Union which include the commitment to provide services for people with chronic illnesses in at least 30 European regions in 2014 in their work plans [18]. The use of Telehealth on a large scale implies attending to a variety of user profiles, usually pluripathological, with needs that evolve over time. It is also necessary to consider different environments used by the actors implicated, with a special focus on patients at home, be they urban, suburban or rural [19]. The development of wearable devices and mobile broadband communications systems make the individual a connection node within Telehealth networks, with the smart phone and tablet terminals as support platforms for Apps and connectivity with biomedical devices and intelligent environments [20].

Mobility is a differential element in Telehealth. It is interesting to note that according to data from the International Telecommunication Union (ITU), the number of subscribers to mobile telephones in 2011 is equivalent to 85 % of the world population [21]. This global digital ecosystem is propitious for the development of social networks for health under the initiative of the patients and professionals themselves beyond the implementations controlled by the health organizations.

A perspective of evolution towards Telehealth systems with a large internal and relational complexity can be deduced from the aforementioned jointly with the interdependency with other systems within the healthcare ecosystem itself and with other external domains such as social services, education, industry, telecommunications, alimentation, urbanism, and the global climate [22, 23].

### Patient safety in Telehealth

More than ten years ago the Institute of Medicine (IOM) of the USA published a report called *To Err Is Human* [24]. Since then efforts to tackle the problem have multiplied on an international scale [25–27]. The growing incorporation of information and communications technologies (ICT) in healthcare has extended the concern for patient safety to eHealth systems [28–30]. Nevertheless, studies into patient safety in the eHealth systems have to date excluded Telehealth [28] perhaps due to the lack of implementation at the time of carrying out these studies. Furthermore, it is illustrative that in the self-descriptions of the cases of good practice, collected in Group B3 of the EIP-AHA in Europe on Telehealth projects, there is no reference to patient safety either in the objectives or in the results of any of the cases [31].

In general, the allusions to patient safety in Telehealth centre on data protection and confidentiality. Nevertheless, as some authors have noted, the questions of safety for Telehealth cover many more aspects [32] such as those related to interoperability [33, 34]. The increased risks involved in the maintenance of data privacy through technical means, human error and malicious actions that may be increased in Telehealth through wireless communications and the variety of environments of use must be taken into account. On the other hand the users might co-create risks as well as co-creating value. There are also social risks because of lack of fairness in access to the new services derived from the digital divide for socially vulnerable and economically deprived populations. There are also perceived psychosocial risks and dependence on technology. On the other hand operating risks in hostile environments arise which are out of the control of healthcare institutions, for example, electromagnetic compatibility, the power supply or extreme climatic or environmental conditions (temperature, humidity, dust). The technology itself may generate health risks such as those related to the biological interaction with electromagnetic radiation associated with mobile communications and sensor networks [35]. Beyond the internal risks of the systems themselves under routine operating conditions, external threats such as biological and chemical accidents, nuclear radiation, industrial accidents and other risk situations might interact with the operation of healthcare services including Telehealth systems [12, 22, 23].

Research into Telehealth has been mainly concerned with proof of the concept and performance of the technological solutions. The work is usually directed at evaluating the use of Telehealth compared with the in situ care as measured in terms of health results. There is currently a great deal of interest in the evaluation of the Telehealth systems [36]. However, there is no similar interest in research into questions of patient safety beyond the aforementioned data security.

Clearly the Telehealth systems share many common interests as regards patient safety and security [29, 37] with other large healthcare computer systems based on ICT. Nevertheless, as a result of their nature, Telehealth systems present significant differential characteristics in aspects such as technology, environment of use, profiles of the people involved, processes, organization and business models. That is why it is necessary to consider the existence of new risks of which there is no significant accumulated experience. Until recently, experience in Telehealth has proliferated in the form of pilots with low-scale implementations and subject to the testing conditions which are not close to real situations in practice. The shift from the pilots to the large systems gives rise to the serious challenge of researching the questions associated with security in the design, implementation and routine operation. Historically, there are multiple examples of large ICT systems, in healthcare and other fields in which the safety measures are added ad hoc after the faults had taken place. It is necessary to change this mentality to take on patient safety from a perspective of anticipation including it in the entire life cycle of the system from the first steps in its conception [38].

The approach to safety in general, not only in health, has evolved from a focus on human error to a perspective of socio-technical systems and developing concepts such as High Reliability Organizations (HRO) [39]. It cannot be assumed that the management of existing clinical risks and the patient safety processes established in traditional health organizations offer the appropriate structure, processes and results to tackle patient safety in Telehealth. Harm to the patient could come about as a result of a number of factors and circumstances. For example, the earthquake, tsunami and nuclear accident at Fukushima imply significant risks to the health of the population. In addition to the effects attributable to the exposure to the radiation analyzed by the World Health Organization (WHO) [40], experts say it is necessary to consider the psychosomatic problems and psychiatric disorders produced by fear, anxiety and depression. On the other hand, a year after the events, the fish caught off the Fukushima coast had levels of cesium of up to 250 times the amount approved by the Japanese government for human consumption. Similar increases have also been detected in the concentration of radioactive elements in agricultural products grown in the area [41]. Nevertheless, it seems that the severest risks to health were caused by the chaotic forced evacuations from hospitals and elderly people’s residencies. The local authorities have estimated that close to 600 deaths were caused by fatigue or through the worsening of the health conditions for critical patients and older people which could have been avoided or alleviated through greater communication and interoperability between the information systems of the different organizations involved [12]. This case dramatically shows the complex nature of the unexpected risks for health and the potential of Telehealth technologies to avoid or reduce the gravity of the impact on the patients and fragile elderly people in similar situations.

Getting back to the healthcare domain itself, according to Adam Darkins, Director of Telehealth of the Veterans Administration program, to guarantee that Telehealth programs maintain or improve the levels of patient safety it is necessary to adopt an approximation of systems in a healthcare transformation [42]. This vision of systems is shared by other experts in the field such as Paul Schyve [43], who in his projection of the future of patient safety in 2025, places emphasis on the need to understand the nature of the complex systems that make up healthcare and adopt the thinking of systems to guide the change.

Research into patient safety requirements for Telehealth systems are explored in this article. Part of the content presented is the result of the work carried out in the PITES Project [44].

## Theoretical framework for the approach to research into patient safety in Telehealth

### Complexity and vision of the systems

Complexity is a characteristic proper to Telehealth systems. Even in its simplest versions, they are socio-technical networks made up of patients, carers, healthcare professionals, biomedical devices, electronic equipment, computers, digital contents and other entities connected to the infrastructures and telecommunications services through which they relate by exchanging information and knowledge at a distance for the care of people. The Telehealth systems operate, in turn, through being immersed in the complex healthcare organizations and are also open to the interaction with other systems that give rise to different behaviors and new approximations in accordance with the contingencies [45].

In Telehealth systems, as in other healthcare structures, the limits are vague and badly defined. The stakeholders in the system (patients, professionals, assistants, technicians, etc.) change and can belong to several systems. On the other hand these people use rules and mental models that are interiorized and cannot be shared with or understood by others. Furthermore, these mental models change over time. For all of these reasons, Telehealth systems challenge a simple comprehensive model. They then become systems characterized by what is known as organized complexity [46]. They are open dissipative systems that include nested complex subsystems and it is presumable that they have the characteristics of open- scale networks [47]. They show non-linear interactions and connections between subsystems and components with negative and positive feedback. Consequently, they are not predictable in the cause-effect relationships, so that small changes at a certain point in the system could bring about large changes in other parts which could consequently give rise to the possibility of numerous faults. Thus, the aforementioned Telehealth systems may be identified as Socio-Technical Complex Systems (CSTS) [48, 49]. The analysis based on CSTS theory has been used previously for the approach to questions related to medical errors by some authors such as Carayon [50].

### Modeled in layers and safety as an emerging property

**Fig. 1:**
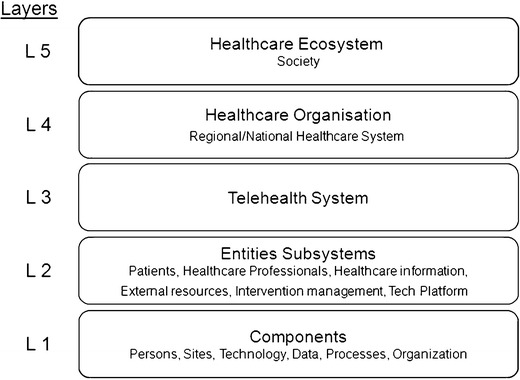
Diagram showing the structure in layers adopted for the analysis of the Telehealth complex socio-technical systems (CSTS). Telehealth system cannot be considered in isolation but at a layer within the hierarchical scale of layers. The lowest layer L1 refers to the components which are used to build-up the upper layer L2 of the entities subsystems. These entities subsystems are constitutive parts of the L3 Telehealth system. In turn the Telehealth system forms part of the layer L4 healthcare organization that is part of the top layer L5 of healthcare ecosystem at society. One layer L (n) can display emergent properties that cannot be discerned from the isolated knowledge of the elements of the lower level L (n-1). Patient safety is one emergent property

Complex Socio-Technological Systems (CSTS) can be modeled as a hierarchy with levels in different layers [38]. The design modeled by the authors for the case of Telehealth is shown in Fig. 1. In agreement with this Telehealth System cannot be considered in isolation but in a level within the hierarchical scale of layers. Each of these layers is explained in detail in Section 3.

A concept relevant to the modeling by layers is that one layer can display properties that cannot be discerned from the isolated knowledge of the parts of the lower level. This phenomenon is known as emerging properties [46, 51]. Security is a property emerging from the different layers of the CSTS. The behavior of the isolated elements of a layer says nothing about the possible behaviors that could take place in connection with the other elements and cannot be explained by a simple addition of separate properties. For example, a biomedical piece of equipment of a patient can be analyzed in an isolated way in a laboratory and considered sound, but when it is used in a Telehealth system, connected to other devices, used by elderly people in uncontrolled environments such as the home, a new type of difficult-to-predict risk situation emerges.

Another significant characteristic of the structure in layers is communication between its components associated to the control necessary in open systems. The control structures are hierarchical by nature and must be established both in the development of the system and their operation. The control implies a higher level by imposing conditions at a lower level. For this an effective communication is required between the layers in such a way that the higher one is able to establish goals, policies and constrictions at the lower level and in turn know the behavior of the components of the lower level. According to Laracy [46], security is an emerging property that is achieved by means of constrictions which allow the questions of safety to become questions of control.

## Characterization of the layers in a modeling of the complex socio-technological systems for Telehealth

### General structure

As has been set out in the previous section of this work, the structure shown in Fig 1 has been adopted. 1 with five hierarchical levels:

- Layer 5: Healthcare Ecosystem in Society
- Layer 4: Healthcare Organization
- Layer 3: Telehealth System
- Layer 2: Entities Subsystems
- Layer 1: Components

These levels are described in more detail in the following paragraphs

### Level 5: healthcare ecosystem in society

The highest level refers to the Health Ecosystem in Society. It is where the “rules of the game” are established, that is, the legal, regulatory and cultural framework that affect the operation of the health organizations that provide the health services to the citizens are established.

The legislative bodies, the administrations and the regulatory agencies operate at this level. Stakeholders, such as institutions and insurance companies, industrial associations, professional colleges, trades unions, patient organizations and other institutions are also situated at this level. International health organizations such as the WHO and OECD, and the standardization bodies (ISO, CEN, HL7 and IHE) are also involved actors.

The macroeconomic and social policies; legal measures; regulations; standards and certification processes related to patient safety are established at this level.

Information on epidemiological data, statistics on events and economic data is received from the lower layer.

### Level 4: healthcare organization

This level corresponds to the action of the organizations providing health care and social services that have the capacity and skills to provide services based on Telehealth. They may be of a private or public nature. It is important to consider the trends towards the integration of health and social care and the inter-operation of public and private services.

Questions specific to this level are the maximization of efficiency in the provision of the services, improving the quality of welfare and adjustment to economic conditions. Each organization is characterized by its institutional configuration together with the formal and informal networks formed by the organizations involved.

The operating rules and regulations for the Telehealth Systems, including quality management and cost/efficiency evaluation, are established at this layer. The requisites refer to patient protection regulations; system certification processes; guidelines for the implementation and operation of the systems; qualification of human resources; rules for reimbursement and economic incentives, as well as measures for the surveillance and notification of adverse events.

In turn, this level receives information from the Telehealth System, situated at a lower level, including data on operation performance, events and changes in the Telehealth systems that might directly or indirectly affect patient safety.

### Level 3: Telehealth system

The Telehealth System is located at this level, that is, a group made up of people, technologies and procedures that support the provision of Telehealth services within the functional and economic framework established by the Healthcare Organization for whom it operates situated at the immediate upper layer. This level is characterized by its organizational structure and operating framework within the Health Organization; healthcare objectives; business model, and functional processes. There is a wide range of Telehealth Systems and different types of Telehealth Systems that could coexist within the same Healthcare Organization e.g. to attend to different types of patient. A Telehealth System may also provide services to different health organizations.

The design of the general architecture is established at this level of Telehealth System together with the articulation of the flows of knowledge and communication between patients, professionals and other actors for the satisfaction of the services that must be covered by the System, for example, to help in the control of oral anticoagulant therapies (TAO); the management of people with hypertension; etc.

The requirements on patient safety for the immediately lower level include best practice guidelines, and both technical and functional requirements for the design and implementation of the different Entities Subsystems.

In addition, it senses adverse events or situations that could lead to risks to patient safety e.g. modifications to the equipment or in the operating conditions of the Entities Subsystems at the lower level.

### Level 2. Entities subsystems

**Fig. 2:**
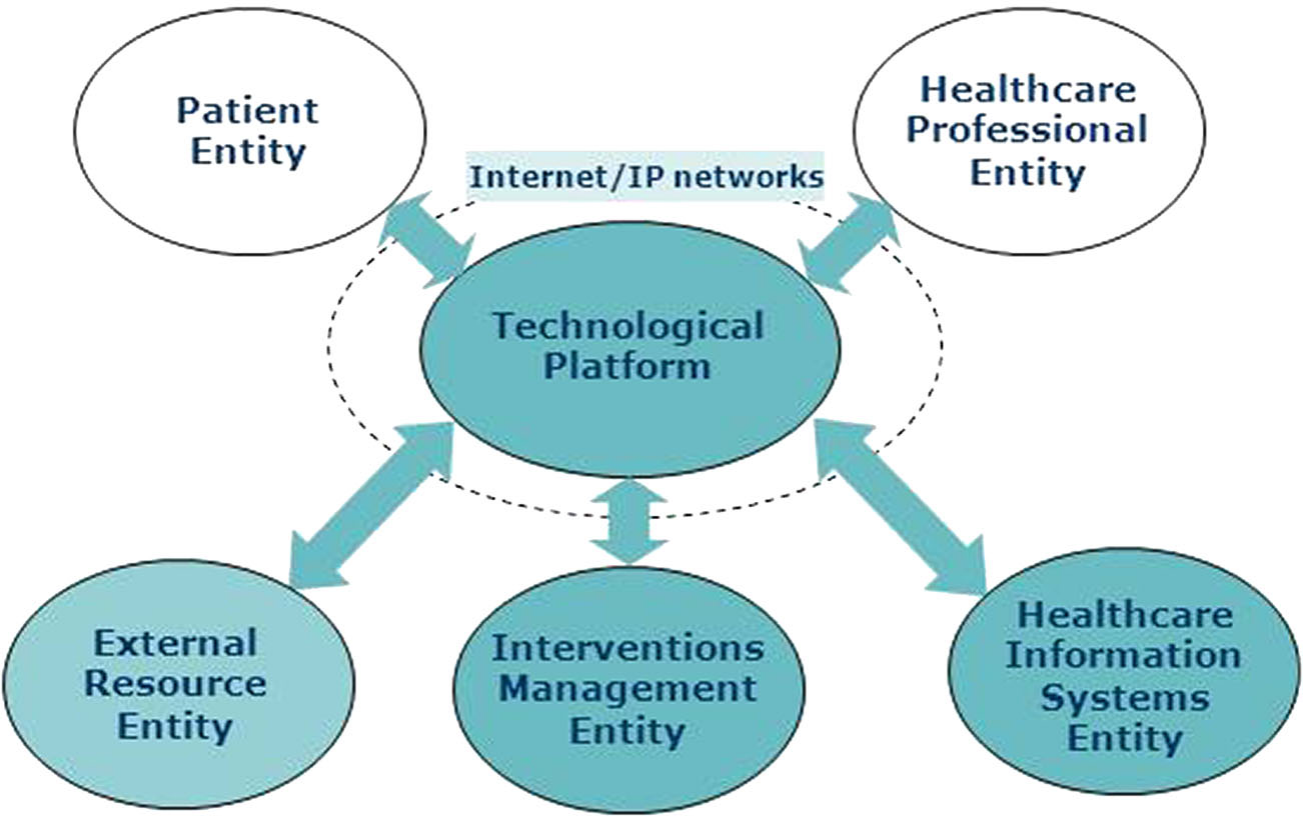
Scheme showing the entities subsystems of a Telehealth system in accordance with the PITES architecture [44]. They are named: patient entity, healthcare professional entity healthcare information systems entity, interventions management entity and external resource entity, all of which are connected by the technological platform using Internet on mobile and fixed networks

This layer corresponds to the group of Entities Subsystems that interact between themselves exchanging information within a healthcare flow process fixed in the layer immediately above that is the Telehealth System. The Entities Subsystems may vary in number and characteristics depending on each specific application case. Its definition depends on the focus and decision of the Telehealth System designer. It is rather arbitrary responding to the logic of the designer dealing with it. There is no standard formal model. The architecture PITES [44], developed in the Telemedicine Unit of the *Instituto de Salud Carlos III* (Carlos 3^rd^ Health Institute), has been used in this work as a reference for the solution architecture as it has application experience in a large number of research projects and trials with chronic patients [34]. According to this model (see Fig. 2), the Telehealth system is made up of the following subsystems: a) Patient Entity; b) Health Professional Entity; c) Interventions Management Entity; d) External Resource Entity; e) Healthcare Information System Entity, and f) Technological Platform Entity. They are described in summary below:

- *The patient entity* encompasses the patient and all of the resources assigned to him or her for the intervention. The patient entity is usually made up of:
  A patient protocol usually consists of periodically carrying out biometric measurements (arterial pressure, weight, pulse, ECG, spirometry, lipid profile, activity, etc.), and replies to questionnaires on symptoms or actions.
  Some biomedical monitoring equipment for personal use (sphygmomanometer, scales, pulse oximeter, thermometer, etc.) to carry out the measurements required by the patient protocol
  Communications equipment to carry out the periodical sending of protocol information. The equipment must be suitable to interact with the interfaces authorized by the technical platform.
- *The healthcare professional entity* represents the perspective of the health professionals and is made up of the series of tools and resources required to carry out the healthcare protocol established by the intervention. In general, it is a set of applications adapted to the specific patient protocols through which the monitoring is carried out including tools and functionalities that make an indirect communication possible with the patient (advice, warnings, etc.). These applications are usually accessible through the Internet with the appropriate access controls. The reply messages to the patients are sent by means of personalized services such as SMS, e-mail, interactive voice systems, etc.
- *The external resource entity* represents any support resource additional to the intervention on the health or community environment. That is health centres, pharmacies, consultations, geriatric residencies, other platforms, etc. The function of this entity is usually to represent any infrastructure that acts as a resource of attending the patients collectively, because it has some type of logistic advantage (economical, location, etc.).
  They can act as external resources, for example:
  Residential homes, in which there is the possibility of attending the patients collectively by means of shared equipment, for example, patients with oral anticoagulation therapy who share the INR monitor and the communications equipment. The interfaces with this type of external resource may be applications based on the Web, designed so that a person responsible for the resource manages the patient collectives.
  Platforms for external monitoring that receive information of specific patient collectives, for example, patients with implantable cardioverter-defibrillator (ICD) monitored from a platform that authorizes the company providing the ICD. In these cases, the interface would be based on specific “middleware” that makes it possible to interoperate with the aforementioned external platform.
- *The healthcare information system entity* represents the human, technical and process resources that make the management and exchange of information between the different entity subsystems possible. For this it makes use of a local Electronic (Tele)Health Record (ETHR) which essentially summarizes the clinical information generated by the patients and health professionals during the Telehealth interventions. This entity also provides the interoperability for communication with the information systems of the Healthcare Organization which the Telehealth System serves. Basically, it involves communication with the Electronic Health Record systems but it may also require communication with the Patient Identification (ID) systems, identification of the professional, and the electronic prescription. This entity (which has been left out of many Telehealth pilots y projects and which has generated islands of information) becomes fundamental for the extended deployment of the Telehealth.
- *The intervention management entity* supports roles and resources that are required to carry out the intervention and that are not available or cannot be carried out suitably either by the healthcare system or the community. The services provided by this entity are of two kinds:
  Support to the deployment of the intervention providing resources to make it possible to train the health professionals, patients, families; the maintenance and management of the equipment; tools for monitoring of the compliance with patient protocols, etc.
  Support methodology of the experimental evaluation study by providing resources for the support methodology of the clinical trials and experimental studies. For example, drawing up and management of the documentation (Case Report Forms), applications for the Electronic Data Capture, services for the centralized randomization, recompilation and analysis of results, among others.
- *The Technological Platform Entity* represents the ICT nucleus that supports the functional interfaces, the coordination of activities and finally the telematic infrastructure that requires the interventions to be implemented during its evaluation. The services provided by the platform are provided mainly through the Internet, digital cellular networks and the public telephone switching network (PTSN).

Every one of the aforementioned subsystems is, in turn, an open CSTS which, as well as being related to the other subsystems of its Telehealth System, do it with other external systems, such as telecommunications services, assurance, transport, information systems, the scientific community, market for healthcare products, etc., which give rise to entries out of control of the Telehealth system itself.

This level sets up the constrictions to the lower level of the Components. They refer to aspects as: requirements on the skills of the professionals; patient information; requirements for the equipment; certification of the equipment and software; quality of telecommunication services; accessibility; codification of data; home premises and environmental specifications.

Feedback information is received from the lower levels of Components for control in the safe design and operation of the Entity Subsystems.

### Level 1 components

This lowest layer includes the following types of Component: C1) Persons; C2) Environment; C3) Technology; C4) Data; C5) Procedures and C6) Organisational Context. They are described below.

- C1) *People*: including individuals, groups, and roles that perform in each “Entity”. For example, the people component of the Patient Entity is typically chronic patients and can include also members of the family or voluntary caregivers.
- C2) *Environment:* including physical site and infrastructures. In Telehealth the medical procedure takes place outside the institutional environment. The healthcare professionals are able to carry out their activities from different locations and in an asynchronous manner, even on the go. For their part, the patients can be seen in their homes or in another convenient place. Other subsystems such as the technological platform can be installed in healthcare institutions, be distributed or, at premises of external provider’s.
- C3) *Technology*: including hardware and software. The different Entity Subsystems use a wide range of technologies from smart sensors linked to wireless networks and telecommunications services, as well as complex software applications. These technologies must cover special requirements. For example the biomedical equipment used by the patients is different by conception and destination of use of the equipment operated by professionals in healthcare institutions. Personal devices require the minimization of size and energy consumption.
- C4) *Data:* this component refers to the different types of data, its codification, how it is saved, formats and rules for exchange in each Entity Subsystem. It includes signals taken by biomedical equipment or environmental sensors, images, audio, voice and free and structured text. Furthermore, it is made up of data compression, filtering, pre-analysis and detection of alerts.
- C5) *Processes:* the way in which things are done (theoretical and real), management models, protocols, report relationships, requisites for documentation, data flow, and relationship regulations.
- 6) *Organizational context*: in which the operation of each Entity sub-system is performed, for example, type of healthcare coverage of the patients (public, private, state assisted); appointment of the professionals; ICT platform provision (centralized, external, in the cloud)

The aforementioned Components serve to characterize the set of Entities Subsystems of a specific Telehealth System. As an example, Table 1 shows the Components of the Entities Subsystems for the case of the Telehealth-based service designed by our group for the follow-up and monitoring of patients treated with oral anticoagulant therapy (OAT) [9]. During the self-testing phase, the patient measures his own INR (International Normalized Ratio) using a portable coagulometer and sends it, together with a short questionnaire, by cell phone to the central station (CS) of the Platform . His general practitioner (GP) accesses the patient data via the Internet and decides the total weekly dose (TWD). The GP has a decision support tool that provides a preliminary TWD on which to base his final decision. A short message (SMS) is then sent to the patient. This is the phase of patient education. In the guided self-management phase, the patient takes responsibility for his TWD, but continues sending his INR and TWD to the central station, where this data can be supervised by the GP at the patient’s request or according to a pre-established protocol. Patients living at home use an individual coagulometer and cell phone but for patients at a nursing home a caregiver uses the same coagulometer and communication device for all these patients participating in the program (External Resource Entity).

**Table 1:** Analysis of the components of the different Entities Subsystems in the case of a Telehealth-based service for the follow-up and monitoring of patients treated with oral anticoagulant therapy (OAT) using PITES “Telehealth system” architecture [9]. The entries in the rows correspond to the entities subsystems, while the entries in columns display the characteristics of the different Components

Subsystems	Components					
	People	Environment	Technology	Data	Processes	Organization
Patient Entity	Chronic patients treated with oral anticoagulant therapy (OAT). Advanced age	Urban domicile Rural domicile Nursing home	Mobile terminal GSM WAP SMS Coagulometer	INR Reply Form Messages Text Voice	Measuring and sending protocol WAP form SMS	Madrid Regional Health System (SERMAS)
Health Professional Entity	General Practitioner (GP)	Medical Office	PC Fixed and mobile Internet connection Software application	TWD Patient folder Evolution curves Alerts Medication Statistics	Care Protocol	Primary Care Madrid Regional Health System (SERMAS)
Interventions Management Entity	Nursing, Sociology, Psychology Technicians	Office	PC LAN Internet Connection Fixed and mobile telephony Multimedia	Patient directory Planning Timetables Statistics Verbal communications Training materials	Protocols and guidelines supervision Patient education Help Desk Quality control	Carlos III Health Institute. Telemedicine Research Unit
External Resource Entity	Formal Caregiver	Nursing home	Mobile terminal GSM WAP SMS Coagulometer	INR Reply form	Caregiver Protocol Caregiver training	Nursing Home Pozuelo (Madrid)
Healthcare Information Entity	Health computing professionals	Research Office	Computer equipment Middleware	Patient Telehealth Records (PTHRS) Patient ID	Patient Telehealth Records (PTHRs) management Data security Technical and semantic communication interoperability with EHR	Carlos III Health Institute Telemedicine Research Unit
Technological Platform Entity	Technologists	ICT Centre	Central station Web services Telecom Infrastructures	All above + ICT management data	Web Services design and implementation ICT Services provision	Carlos III Health Institute Telemedicine Research Unit

*GSM* Global System for Mobile *Communications*; *WAP* Wireless Application Protocol; *SMS* Short Message Service; *INR International Normalized Ratio* (*INR*) for coagulation testing; *TWD* Total Weekly Dose; *PC* Personal Computer; *LAN* Local Area Network*; ICT* Information and Communication Technologies; *ID* Digital Identification

**Fig. 3:**
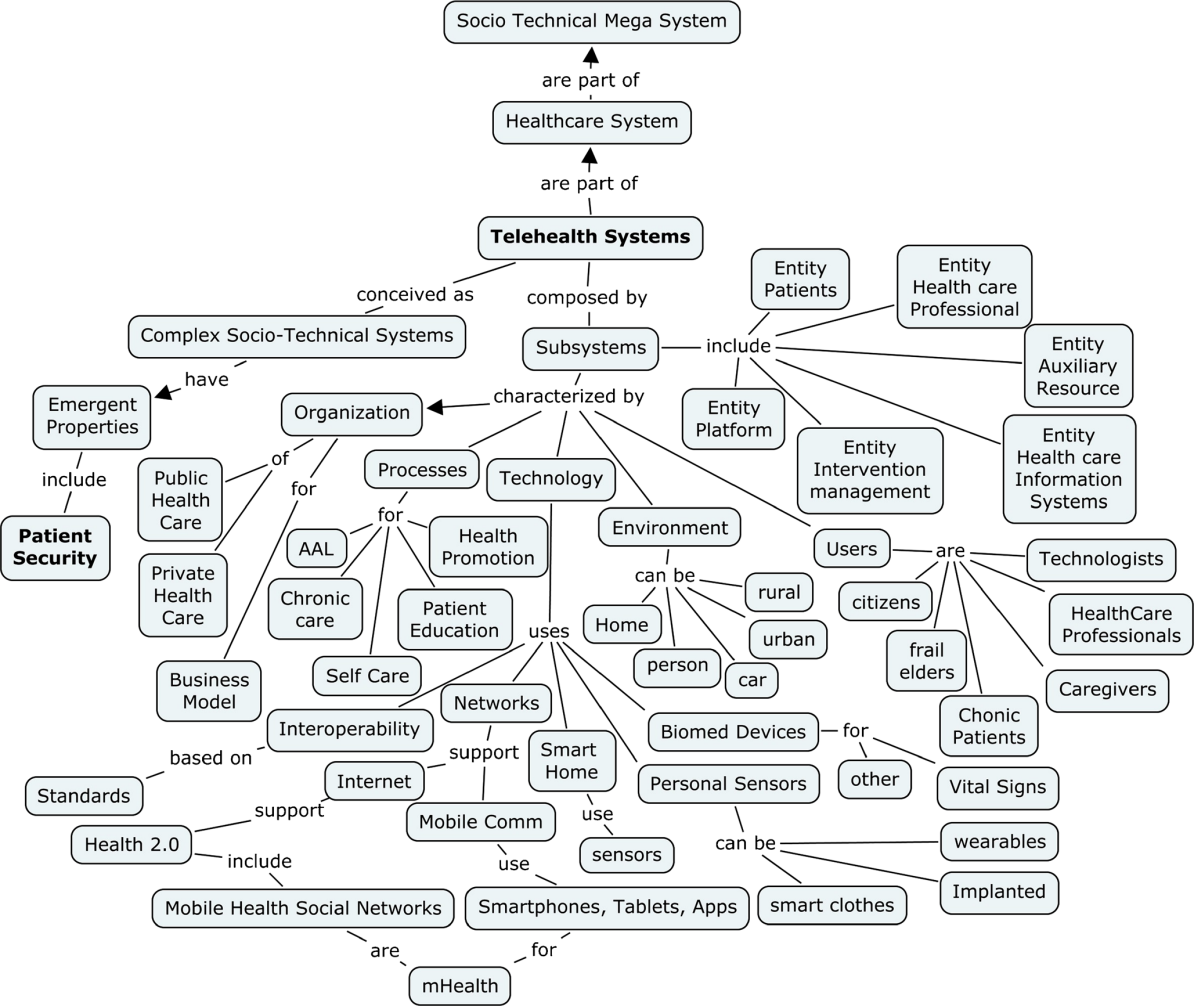
Conceptual map of Telehealth Systems drawn up by the authors. It is based on the adopted layers structure, detailing the entities subsystems entities and their components concerning users; environment; technology; processes, and organizational context. it is a tool to facilitate the structured analysis and the synthetic vision of the constitutive elements of the lower levels of the Telehealth systems

Similar tables can be constructed for each different case of Telehealth application to be analyzed. To facilitate the analysis the authors have constructed a conceptual map that is shown in Fig. 3. It reflects the structure of layers emphasizing the characterization of the Entities Subsystems and their Components. It is a tool to facilitate the logical and structured organization of the different constitutive elements and their links. For example, in the presented case of the application in oral anticoagulation therapy (OAT), it has served as a guideline for the systematic analysis of the Components of each Entity Subsystem as displayed in Table 1. Furthermore, it provides a framework for the incorporation of new elements which could give arise with experience from other cases concerning the typology of the users, environment of use, etc.

## Identification of areas of research for safety in Telehealth

The proposed research lines are displayed in Table 2. The core set of research domains is derived from the model of hierarchical layers for CSTS and the conceptualization of patient safety as an emergent property of each layer. They exhibit a greater degree of abstraction as it ascends in the levels, which translates as different scopes and focuses on the needs for research. In addition, the list includes a research line on theoretical aspects as well as another one on research methodologies and tools.

**Table 2:** Proposal for lines of research into patient safety in Telehealth systems, indicating the objectives and a list of research topics for each of them

Research lines/Analysis level	Objectives / Control parameters	Research Topics
Theory of CSTS Engineering in Telehealth	Scientific reference framework	CSTS Engineering methods and tools Dynamic Cognitive Networks Mobile Social Networks Resilience in Telehealth Systems
Patient Safety at the level of Healthcare Ecosystem in Society	Quality of Life (HLY^a^) Epidemiological data Macro-economic data	eHealth Governance Legal and organizational interoperability Patient empowerment policy Vigilance policy for patient safety in Telehealth Safety standards Certification Legal and ethical issues Holistic approach for patient safety
Patient Safety at the level of Healthcare Organization	Health outcomes Quality of Telehealth services Data of adverse events Economic data on Telehealth safety investments and operational costs. Costs related to adverse events Compliance with laws and regulations	Requirements Engineering for Patient Safety in Telehealth Implementation of vigilance policy Evaluation methodologies and tools for patient safety in Telehealth Organizational and semantic interoperability Integrated Models for patient safety at healthcare organizations
Patient Safety at Telehealth System level	Data of system performance Quality of Telehealth services Data on adverse events Micro-economic data on patient safety implementation and operation Compliance with standards and guidelines	Systems design for safety Telehealth systems standardisation Semantic interoperability Organizational interoperability Safety management in Telehealth Vigilance tools
Patient Safety at Entities sub-systems level	Adverse events detection and report Adherence of patients and professionals to protocols and guidelines Equipment and software compliance with safety and security standards Physical and environmental conditions at patient’s home Information on components change and performance	Secure patient and professional identification Tools and methods to measure and manage caregivers workload Decision support tools for healthcare professionals Accessibility and user interfaces Safety of personal devices and wearable Home devices interoperability Cloud safety for Telehealth applications Apps safety Non-invasive surveillance techniques Behaviour monitoring and data analysis Home systems reliability Energy issues Electromagnetic Compatibility at home
Methodologies and tools	Support technologies and techniques	Data mining and Analytics Geographical Information systems (GIS) Digital simulation Modeling languages Methods and tools for Telehealth system redesign

^a^ *HLY* Healthy Living Years

The description, following the same order of the rows at the Table 2, is as follows:

i) In first place, research is proposed into theory of CSTS Engineering applied to patient security in Telehealth covering topics as Dynamic Cognitive Networks; Mobile Social Networks, and Resilience in Telehealth Systems. This line aims to provide theoretical foundations for systemizing the knowledge and the interchange of experience within a common reference framework.
ii) Research on Patient Safety at the level of Healthcare Ecosystem in Society. It is concerned on improving quality of life of the population, measured for example in Healthy Living Years (HLY); epidemiological data about patient safety, and macro-economy. Topics of research in this line include eHealth governance; legal and organizational interoperability; patient empowerment; vigilance systems for adverse events; standards; certification of platforms; legal and ethical issues, and holistic approach for patient safety in Telehealth.
iii) Research on Patient Safety at the level of Healthcare Organization. This line address issues as health outcomes; quality of Telehealth-based services; prevention of adverse events; implementation of standards; patient safety management; efficiency and operational costs. Research topics include engineering on patient safety requirements; implementation and management of adverse events vigilance for Telehealth; integrated models for patient safety at healthcare organizations, and methods and tools for evaluation of patient safety in Telehealth systems.
  The implementation of Telehealth services requires a lot more than just adding a new process to an existing healthcare organization. Rather, a redesign of the system is required, with new functions and new organizational environments. Thus, at this level the research on organizational and semantic interoperability takes on a major relevance.
iv) Research on patient safety at the level of Telehealth Systems. This line focus on issues as Telehealth-based services performance; quality of the services; data on adverse events; micro-economy of implementation of patient safety measures, and compliance with standards and regulations.
  The list of research topics includes systems design for safety; Telehealth systems standardization; archetypes for records of patient safety in Telehealth; organizational interoperability of Telehealth within healthcare organisations, and processes for patient safety vigilance.
v) Research on patient safety at the level of Entities sub-systems. Patient safety at this level is an emerging property from the constitutive Components of each entity sub-system, that is: people, environment, technology, data, processes, and organizational context. Therefore this research line address issues such as local adverse events prevention, detection and report; adherence of patients to protocols; physical and environmental risks at patient home; performance of healthcare professionals; equipment and software compliance with safety standards and regulations, and control of technological evolution and operation routines.
  According to the above, the list of research topics covers a wide range: Secure Patient and Professional Identification; tools and methods to measure and manage caregivers workload; decision support tools for healthcare professionals; user accessibility; safety of personal devices and wearable sensors; devices interoperability; cloud safety for Telehealth applications; Apps safety ; non-invasive surveillance solutions ; behaviour monitoring and data analysis; home systems reliability and electromagnetic compatibility.
vi) Finally, the set of lines of research is completed with research into methods and tools. The list of research topics include: modeling languages; digital simulation tools; data mining and Analytics; Geographical Information Systems (GIS); modeling languages and methods and tools for system redesign in Telehealth.
  The above proposal is the result of a reflection on the needs for research directed at patient safety in Telehealth under the conceptual framework we have developed in Sections 2 and 3. The list of identified lines and subjects responds to the current analysis and logically it is open to revision. It has to be borne in mind that the dynamic evolution proper to Telehealth systems, subject to a significant technological and social change, supposes the necessary adaptation of the research to this envisaged evolution. From this perspective, the focus of the research must be open to the entire life cycle of the system in an interactive manner and integrating it into risk management.

## Discussion

The ICTs offer a great potential for improving patient safety when facilitating the acquisition, communication and analysis of information, which facilitates the decision-making process and its implementation [3, 13, 34, 52]. Furthermore, the Telehealth networks, through direct communication with the patients allow the early detection of health problems and alert the Public Health Services of adverse events linked to medication, foodstuffs, climatic conditions, epidemics and other risk situations [5, 6, 22, 23]. On the other hand they facilitate the integration with the social services and allow the carrying out of the concept of Ambient Assisted Living (AAL) so that elderly people are able to live independently in their own homes for as long as possible thus delaying their having to live in nursing homes [53, 54]. Furthermore, Telehealth allows innovative solutions for the control of risks and protection of the people in pandemic situations, like that proposed by L. Kun [55].

However, it is also known that systems based on ICT introduce new sources and types of risk to the patient safety as it can have a significant bearing on the increase in systematic complexity [28, 29].

Patient safety is a critical aspect in the extended acceptability and adoption of the new models for the provision of care supported by Telehealth. The effective development of Telehealth depends on the capacity to get the confidence of the actors involved as it is necessary for the systems to present the property of safety as well as security, correct working, availability, trust, performance, and privacy [56]. Cooper [57] has identified 23 "gaps" in research into patient safety in the USA although these results have been obtained from a limited number of reference projects. The basic research carried out to understand the causes of medical errors and faults in the system are encountered between the aforementioned gaps. Clearly in the case of Telehealth there is a lack of historic knowledge on the risks and faults in operation.

There is a wide range of approximations to security in large ICT systems. The most common technique is that of applying “good practices”. This approximation is usually implemented systematically and reduces or eliminates only the most obvious vulnerabilities [58]. Clearly, this is insufficient in the domain of Telehealth which is under construction. Experience shows the difficulties and economic, social and political costs of introducing designs effectively *a posteriori* to improve security. From there, the importance of considering patient safety during the entire life cycle of the Telehealth systems from the earliest steps instead of waiting for problems to arise [38].

Among the few proposals to tackle patient safety in ICT systems for health from a socio-technical perspective, the so-called Interactive Socio-Technical Analysis (ISTA) developed by Harrison et al. can be found [37]. It emphasizes the recursive processes that appear when a new ICT health application is introduced and generate second-level changes in the social system giving rise to unintended consequences. Also, by using the CSTS vision, SEIPS (Systems Engineering Initiative for Patient Safety) has been proposed by Carayon et al. [59]. In SEIPS, the healthcare systems are conceptualized as work systems in which the people carry out multiple tasks using several tools and technologies in a physical environment and under specific organizational conditions. The interactions of the system influence the care processes and the results in the patients. This approach is shared in our layers model at the “Entities Subsystems” level. Recently, Carayon [50] has proposed an agenda for the analysis of socio-technical systems in healthcare which includes safety but does not make reference to Telehealth systems.

Other contributions in the literature refer to the potential of transferring the health field methodologies developed in other industries such as aerospace [60]. There is, certainly, a great potential in taking advantage of the knowledge and tools developed in other fields for the design of secure socio-technological systems. An example is the STS-ml language developed at the University of Trento for the modeling of security requirements [61].

Patient safety is a global issue that affects countries at all levels of development. In particular, patient safety in Telehealth has a global dimension inasmuch as it is directed at providing solutions to global health problems (ageing, non-transmissible illnesses) and its development is maintained in the context of a global digitalized world, beyond developed countries [62]. In this sense, the development that mHealth is undergoing is notable in developing countries showing a great potential for the future [63].

## Conclusions

In recent years we have been seeing the growing adoption of Telehealth in the healthcare industry to improve the quality and efficiency of the attention to chronic patients and the elderly, for the protection and promotion of the health of our citizens. Furthermore, from a wider perspective, it has a great potential in the application of healthcare crisis management brought about by pandemics, acts of terrorism, catastrophes or industrial accidents at a local, national and global level. The development of Telehealth is boosted by the growing technological capacity in mobile broadband communications, the Internet, wearable sensors, biomedical devices, social networks, cloud computing, big data, automation of processes, GIS, and many other hard and soft technologies incorporated into the components and subsystems used.

This involves considering a new situation in many technological and human aspects in relation to other ICT applications for Health, where patient safety and security are critical aspects.

Research into patient safety in the deployment of large-scale Telehealth systems is faced with a series of significant challenges. The main is that the transformation processes of healthcare systems to improve the quality of care for chronic patients and fragile elderly people based on Telehealth involves a great cultural change in the Healthcare sector [64]. Those responsible for the design, implementation and operation of Telehealth systems would need to adopt a vision of systems and understand the emerging nature of a series of properties among which safety is encountered.

In this article a theoretical reference framework has been explored for research into patient safety in Telehealth based on its conception as Complex Socio-Technical Systems (CSTS) in which people and technologies interact and numerous contingencies are faced which cannot be totally anticipated. The carrying out of prospective research approaches, as proposed by the authors in Table 2, would contribute to creating evidence on the management of patient safety in Telehealth which may be effective and sustainable in a way similar to that tackled in other sectors [38, 46, 65]. Furthermore, it would allow new perspectives to be acquired for the design of Telehealth systems of the future opening up ways leading to innovation for the improvement in the quality of healthcare services, but also in the technological and engineering innovation at all levels, from the elementary components to the emerging socio-technical mega-systems in our society. Under the current scenario the incorporation of a holistic and global vision is necessary considering the interoperability with a wide range of systems of different sectors, beyond health care itself, and the nature of the emerging property of safety in the so complex socio-technical healthcare ecosystem.
